# Cold-Season Epidemic Dynamics of COVID-19 in Two Major Metropolitan Areas in Greece: Hypotheses and Implications for Public Health Interventions

**DOI:** 10.3389/fmed.2022.861185

**Published:** 2022-05-30

**Authors:** Spyros Sapounas, Angeliki Bistaraki, Edison Jahaj, Anastasia Kotanidou, Pagona Lagiou, Gkikas Magiorkinis

**Affiliations:** ^1^National Public Health Organisation, Athens, Greece; ^2^Department of Nursing, School of Health Sciences, Hellenic Mediterranean University, Crete, Greece; ^3^First Department of Critical Care Medicine & Pulmonary Services - Evangelismos Hospital, School of Medicine, National and Kapodistrian University of Athens, Athens, Greece; ^4^Department of Hygiene, Epidemiology and Medical Statistics, School of Medicine, National and Kapodistrian University of Athens, Athens, Greece; ^5^Department of Epidemiology, Harvard T.H. Chan School of Public Health, Boston, MA, United States

**Keywords:** COVID-19, epidemic, vaccines, prevention, SARS-CoV-2

## Abstract

Many respiratory viruses, including coronaviruses, follow seasonal transmission dynamics. Analyzing the social and environmental mechanics of the emergence of SARS-CoV-2 over the first cold season provides insight into designing targeted interventions. We analyzed all fully anonymized SARS-CoV-2 case data in two metropolitan areas, Attika and Thessaloniki, diagnosed between September 1st and December 31st, 2020. The emergence of the second wave in Greece occurred in October-November. SARS-CoV-2 diagnoses in Thessaloniki increased quasi-exponentially in mid-October, coinciding with the increase in the proportion of diagnoses in young people aged 18–39. The same pattern was observed in Attika with an almost 2-week delay, even though Attika had a higher prevalence of cases throughout summer until the second wave. Crucially, the nighttime temperature in Thessaloniki dropped below 18°C 3 weeks earlier than that in Attika. Epidemic growth was independently associated with the proportion of cases attributed to the 18–39 age group as well as with the drop in nighttime temperature below 18°C in both metropolitan areas but with a time difference. This pattern can be explained by a shift of nighttime entertainment activities from open-air to closed spaces, which occurs as nighttime temperature drops. Vaccination of young individuals can be crucial in decelerating the cold-season dynamics of SARS-CoV-2.

## Introduction

The global emergence of SARS-CoV-1 in 2003 was successfully contained (1); however, several cases occurred in China during winter, even though there was no evidence of endemic continuity of the epidemic (2). While the exact source of SARS-CoV-1 cases during winter in China remained elusive, this re-emergence raised the concern that SARS-CoV-1 could follow a seasonal pattern similar to influenza or other coronaviruses (3, 4). Our understanding of seasonal patterns of respiratory disease has developed over the years, and several hypotheses have been explored, including environmental factors (4) and host susceptibility (5). Global cases of COVID-19 climbed rapidly from October until November 2020 and then slowly within December 2020 until January 2021 (6), resulting in the largest COVID-19 death toll within 2020. Understanding the dynamics of COVID-19 emergence in the cold season would allow the development of targeted and timely interventions.

The first wave of COVID-19's pandemic in Greece had a relatively low impact. Until June 30th, 2020, 331 cases per million residents and 18.5 deaths per million residents were recorded (7), much lower than the average rates observed in most European countries. This relative success in controlling the epidemic during the first wave can be at least partially attributed to the strict national lockdown with a stay-at-home order that was imposed on 23rd March, just 11 days after the first recorded death. The lockdown was reverted on 4th May 2020 with a gradual lift of social distancing rules. Before this first national lockdown, there was a period of 13 days during which measures of social distancing were imposed, such as school closure and banning of leisure activities, dining and public gatherings.

Between May and the end of June 2020, there was a gradual reversal of public health measures that included the reinitiation of schools, retail shops and dining. Between July and the end of August, there was a period of mild resurgence of cases that slowly continued in September. During this period, the epidemiological situation was assessed on a region-by-region basis with targeted measures of social distancing taking place at the local level with mandatory mask wearing in closed spaces and public transport throughout the Greek territory.

Greece's population is estimated at the level of 11 million based on census data in 2011, with the largest proportion residing in the 2 main metropolitan areas, Attika and Thessaloniki. Attika is located in the middle to southern part of the country, while Thessaloniki is located in the northern part. In mid-to-end October, there was a rapid increase in cases in Thessaloniki that resulted in a series of targeted public health measures (curfew, early closure of bars and restaurants) and ended up imposing a local lockdown with stay-at-home orders at the end of October. Subsequently, an increase in cases was observed in Attika and other regions; thus, a nationwide lockdown with stay-at-home orders was imposed on November 7, 2020.

Here, by analyzing the epidemiological profile of cases that emerged in Thessaloniki (Thessaloniki Metropolitan Area) and Attika (Athens Metropolitan Area) between September 1st 2020 and January 1st 2021, we explore potential hypotheses regarding the dynamics of COVID-19 emergence in the cold season. We show that the exponential increase of the second wave in Greece coincided with the switch of activities from open-air to closed spaces due to the drop of temperature and was led by increased transmission within mid-age groups. Our analysis recorded the summer-to-winter switch of SARS-CoV-2 epidemic dynamics and explores hypotheses for designing timely public health interventions.

## Methods

### Data

**Figure 1:**
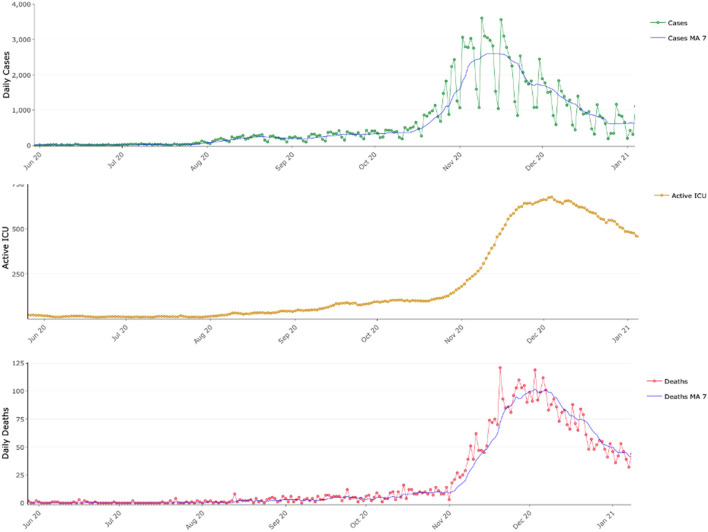
COVID-19 epidemic dynamics in Greece between June 2020 and January 2021. Daily reported COVID-19 cases with their 7-day moving average (MA 7), active COVID-19 intensive care unit beds and daily reported COVID-19 deaths along their 7-day moving average (MA 7).

The measures for processing personal data for the protection of public health are ruled in the Act of Legislative Content of 14.03.2020 (FEK 64/14-03-2020) that was updated by the Greek Law 4764/2020. More specifically Article 5 of the Act of Legislative Content declares that fully anonymized data of the Contact Tracing Program of the Hellenic Civil Protection may be used for research purposes after 31.12.2020. In accordance with the law and following permission request to the Contact Tracing Program of the Hellenic Civil Protection to perform a comparative study of the epidemics in Attika and Thessaloniki, approval along with fully anonymized COVID-19 case data were provided for secondary analysis by the Contact Tracing Program of the Hellenic Civil Protection on 11.01.2021. Moreover, we used published reports of the National Public Health Organization between September 1st 2020 and December 31st 2020 (Figure 1). Contact tracing was implemented from the beginning of the pandemic in Greece (early March 2020) when low transmission was occurring. Greece adopted a standard method of contact tracing (8), using telephone calls to communicate with infected people, isolate them and identify their contacts while inserting all relevant information into an electronic platform. Only confirmed cases were contact-traced and not suspected cases. The electronic database was designed to comply with all the security protocols and national regulations on the use and protection of personal data, and each tracer had his/her own unique password to enter the database. For the purpose of this study only anonymized data were used according to Article 5 of the Act of Legislative Content as secondary for the purpose of statistical analysis, and the article presents aggregated data with no person identifiable information. Historical temperature data were obtained from an internet public source (9). Attika includes the Athens Metropolitan Area with co-ordinates (38.0458° N, 23.8585° E), while Thessaloniki includes the Thessaloniki Metropolitan Area with co-ordinates (40.6401° N, 22.9444° E).

### Mathematical Modeling

We estimated the number of effective cases at time *t* (*E*_t_), that is, the number of people diagnosed with SARS-CoV-2 who can potentially transmit, assuming a period of infectivity of 10 days (10–12). Estimates of effective reproductive number (*R*_t_) for SARS-CoV-2 are subject to multiple sources of bias (13), including testing rate, when cases are used to estimate *R*_t_, and age distribution, when hospitalization and death rates are used to estimate *R*_t_ (14). An estimate of the basic reproductive number *R*_0_ of an epidemic can be calculated by assuming a deterministic susceptible-infected-removed model (SIR) (15):


$$\begin{array}{l} R_{0}=\frac{1}{\left(\gamma +\mu \right)t}\ln\frac{N\left(t\right)}{N\left(0\right)}+1 \end{array}$$

where *N*(*t*) is the number of people infected with SARS-CoV-2 at time *t, N*(0) is the number of people infected with SARS-CoV-2 at the beginning of an exponential growth phase, γ is the rate of people who cease to transmit and μ is the rate of death. Such an equation can be used to estimate epidemic growth assuming that we can fit an exponential curve between time points t and 0.

As a surrogate marker of epidemic growth, we estimate an effective growth factor *g*_f_ of the epidemic by fitting an exponential growth curve on effective (observed) cases observed 10 days apart. We assume that the population death rate is negligible compared to the recovery rate, and thus, *g*_f_ can be estimated by means of the number of effective cases observed 10 days apart (i.e., at time *t* and *t*-10) by means of formula (1) as follows:


$$\begin{array}{l} g_{f}=1+ln\left(\frac{E_{t}}{E_{t-10}}\right) \end{array}$$

In practice, *g*_f_ is a marker of epidemic growth and can be realized as the effective reproductive number of a model population growing following deterministic SIR and exponential growth between time points t-10 and t with respective prevalence of *E*_t−10_ and *E*_t_.

## Results

### Emergence of Second Wave in Attika and Thessaloniki

**Figure 2:**
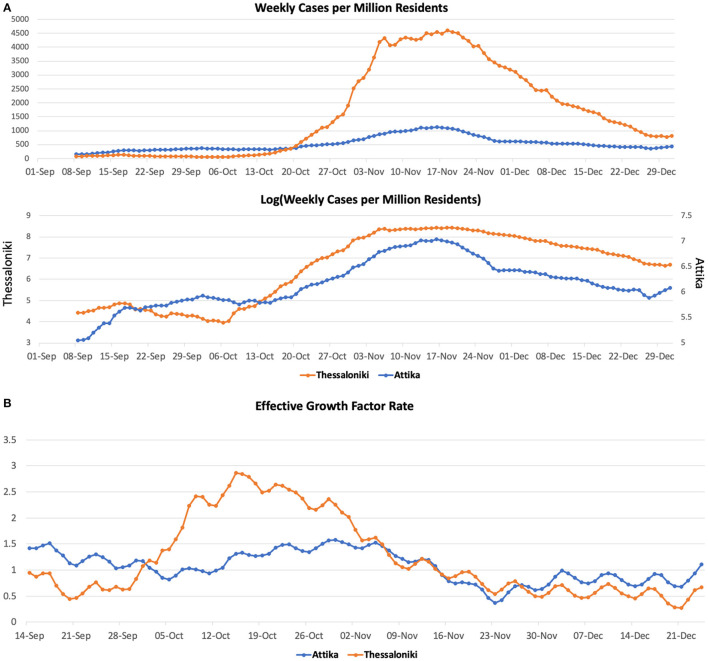
COVID-19 cases **(A)** and effective growth factor **(B)** in Attika and Thessaloniki between June 2020 and January 2021.

SARS-CoV-2 weekly cases per million residents in Attika remained at the level of 300 from early September until mid-October and then gradually increased 3-fold until mid-November (Figure 2A). These dynamics are captured by the effective growth factor rate (*g*_f_) estimates (Figure 2B), which declined in September from 1.4 to below 1 and then gradually increased to 1.6 until early November.

In Thessaloniki, SARS-CoV-2 weekly cases per million residents within September remained at the level of 50. We then observed a 2-fold increase within the first 10 days of October, a 4-fold increase by 20th October and a 40-fold increase by the end of October. These dynamics are captured by the effective growth factor rate (*g*_f_) estimates (Figure 2B), which remained below 1 in September, then rapidly increased to 2.9 until mid-October and then declined to below 1 within the first 10 days of November.

### Second Wave Dynamics Coincided With a Switch in the Proportion of Cases Aged 18–39

**Figure 3:**
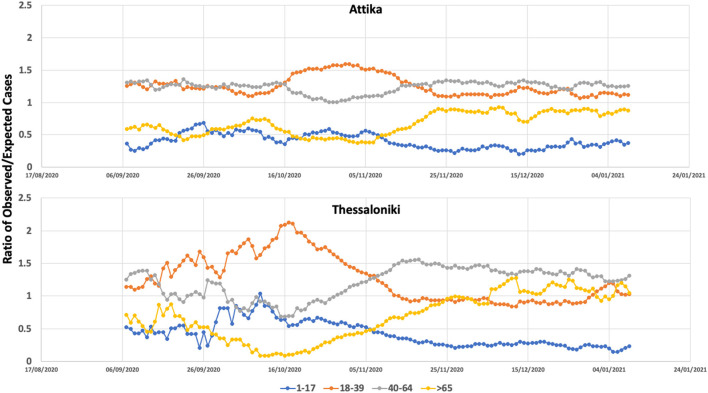
Ratio of observed versus expected number of COVID-19 cases within the age groups of 0–17, 18–39, 40–65, and >65 in Attika and Thessaloniki between September 2020 and January 2021.

Between September 2020 and November 2020, the age groups that had an overall and continuously higher incidence of SARS-CoV-2 in Greece were the middle age groups (18–39 and 40–65). On the other hand, the younger and oldest age groups had proportionally fewer cases. As described above until the end of September, cases in Thessaloniki were continuously low, but the situation changed rapidly between 10th and 20th October, when cases started to increase in a quasi-exponential fashion. The rapid change in transmission dynamics coincided with an ~100% increase in the proportion of cases within the age group of 18–39, which resulted in all remaining age groups having lower-than-expected incident cases (Figure 3). From mid-October until end-October, a series of social distancing restrictions (earlier night-time closure, curfews) were imposed that were followed by a significant drop in *g*_f_ and the proportion of cases within the 18–39 age group. On November 7th, a second national lockdown was imposed. On November 7th, the proportion of 18–39 dropped at the second highest position, and by 15th of November, the observed cases were very close to the expected number of cases for this age group. The proportion of cases within the two older groups (40–65, >65) increased as the proportion of 18–39 declined and remained higher than expected until the end of December.

A similar age pattern was observed in Attika, with an almost 10-day time difference. A significant increase within the 18–39 age group was observed, although in Attika, the increase was 50% compared to the baseline, much lower than Thessaloniki. These 18- to 39-year-old dynamics were most likely decelerated as a result of the social distancing restrictions that were imposed and most importantly as a result of the second national lockdown. Similar to Thessaloniki, the proportion of the two older-aged groups increased as the proportion of 18–39-aged cases decreased, although not at the same level. Thus, in Attika, the older-than-65 group did not increase over the expected number of cases.

### The Switch in Epidemic Dynamics Coincided With a Below 18°C Drop in Nighttime Temperature

**Figure 4:**
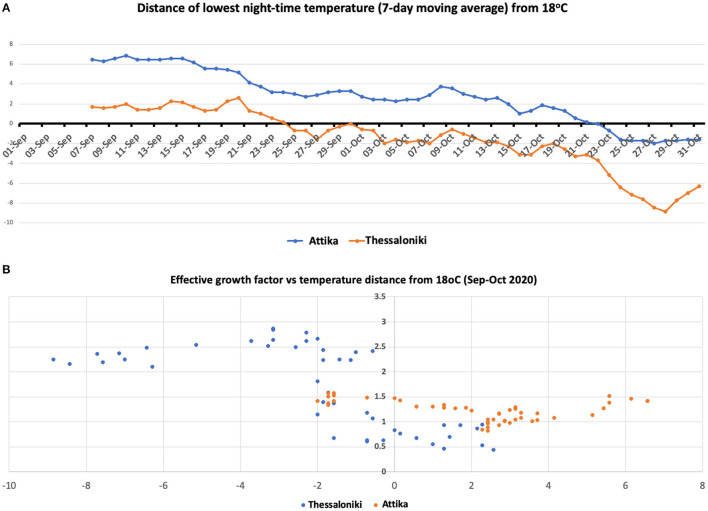
**(A)** Seven-day moving average temperature difference from 18°C in Attika and Thessaloniki. **(B)** Scatterplot of the effective growth factor and moving average temperature difference from 18°C in Attika and Thessaloniki.

Thessaloniki is ~300 km north of Attika. The climate between these two areas is quite different, especially during nighttime, with a 4–5 degree difference at the temperature low point at night (Figure 4). The high season for both cities coincides regardless of the climate difference. We considered the threshold of 18°C, as it has been set by the WHO as the minimum temperature for safe and well-balanced indoor environments during cold seasons (16). The lowest nighttime temperature dropped below 18°C by the end of September in Thessaloniki, while in Attika (Athens), the drop occurred within the last week of October. The scatterplot between *g*_f_ and distance from 18°C showed that *g*_f_ increased when the temperature dropped below the 18°C zone, with the largest effect being observed when the temperature dropped below 16°C. The drop in nighttime temperature results in an indoor transfer of nighttime activities, which are almost exclusively relevant to entertainment (bars, clubs), but during the warm season, they are exercised outdoors. Such entertainment activities are mostly attended by the 18–39 age group, which is in line with the observation that their proportion in the number of cases increased in Thessaloniki and Attika. The phase difference in the switch of 18–39 age group proportion between Thessaloniki and Attika also fits the pattern of temperature drop.

### Second Wave Transmissibility Was Led by the 18–39 Aged Group

**Figure 5:**
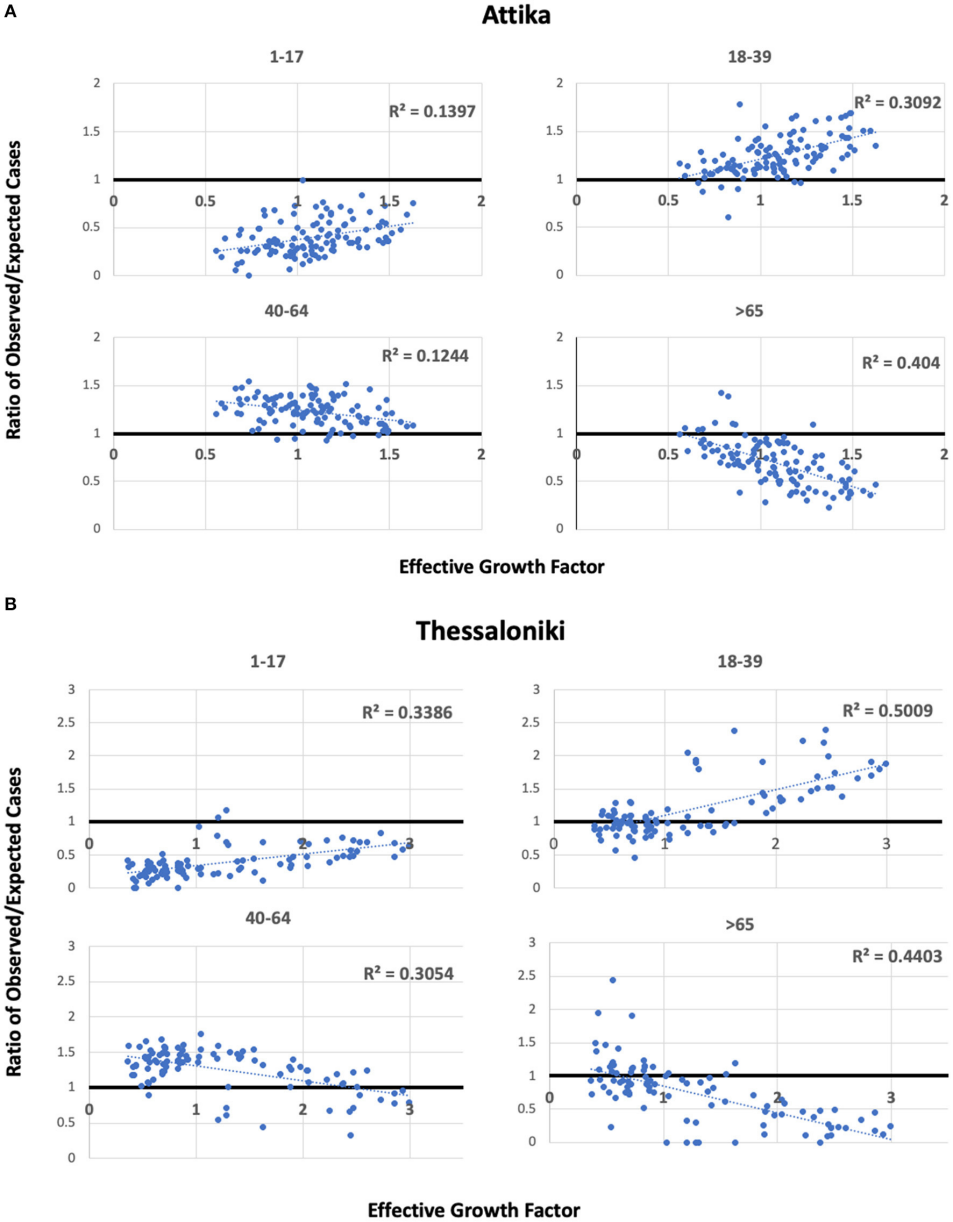
Scatterplots of observed/expected cases per age group and the effective growth factor in Attika **(A)** and Thessaloniki **(B)** along linear regression lines and estimated Pearson's *R*^2^.

To explore the hypothesis that the 18–39 group lead transmission in Thessaloniki and Attika, we built scatterplots of the observed/expected ratio and the effective growth rate (Figure 5). For the two younger age groups, we found positive correlations of the effective growth rate with the higher-than-expected proportion of cases in the 18–39 group and the lower-than-expected proportion of cases in the 1–17 group. For the two younger age groups, negative correlations of the effective growth rate were observed with the higher than the expected proportion of cases in the 40–64 group, whereas the lower than the expected proportion of cases in the >65 group. These patterns were observed in both Thessaloniki and Attika, suggesting that the underlying dynamics of the emergence of the second wave were similar. This pattern suggests that there was an amplification of the epidemic as a result of increased transmission within the 18–39 group. It also suggests that the youngest age group was acting passively in ongoing dynamics and that their proportion increased as a result of increased community transmission, an observation supported by previous research. On the other hand, the 40–64 age group acted as an intermediate with mostly more than expected cases, although their proportion was negatively correlated with the increase in community transmission. Finally, the older than 65 group acted as a sink, and their role was rather negative in community transmission. The age patterns of transmissibility were independently observed in Attika and Thessaloniki, although in Thessaloniki, the association was stronger in all age-group categories.

## Discussion

The epidemic of SARS-CoV-2 in Greece during the second wave had a major impact in the northern part of the country, with the epicenter being the second largest metropolitan area, Thessaloniki. The number of cases in Thessaloniki rapidly increased within October, while previously, the epidemic was well-moderated by a combination of local social distancing interventions that kept the number of cases below 100 per million residents for more than 3 months. In Attika, an increase in cases was also observed with a time difference of ~10 days that was decelerated as a result of the national lockdown imposed on 7th November. We thus aimed to explore the ecology of the rapid increase in transmissibility within the second wave by studying the association of the age structure of the population and nighttime temperature.

We found that the rapid increase in transmission during this period was associated with infections among those aged 18–39, suggesting that superspreading in the second wave was initiated within this age group. Crucially, this association was independently observed in Attika and Thessaloniki. An increase in the proportion of 18–39 was first observed in Thessaloniki and was followed in Attika after approximately 10 days. The case load was significantly lower in Thessaloniki than in Attika, suggesting that the driver of transmission was not a prewave higher prevalence that resulted in this phase difference. We showed that the nighttime temperature in Thessaloniki dropped below the comfort level ~1 month earlier than that in Attika. This drop in nighttime temperature in Greece is associated with entertainment activities moving from open space to indoors, activities that are mostly organized and attended by the 18–39 age group. The major difference between these activities and other work or entertainment activities is the lack of face-mask protection and minimal adhesion to social distancing rules.

As a result of the increasing number of cases in Thessaloniki, a series of interventions took place that targeted night-time entertainment activities, including reduction of opening hours on 9th October, social distancing measures and a night-time curfew at 00.30 on 23rd October, and finally a lockdown with “stay-at-home” orders on 30th October. During this time, the effective growth rate dropped significantly, and the proportion of cases belonging to the age group of 18–39, suggesting that preceding public health interventions successfully decelerated the spread of the epidemic.

The second wave of the epidemic in Attika had the same age footprint as that in Thessaloniki but was followed ~10 days later, even though the prevalence was 5–6 times higher in Attika. The rapid increase in cases in Thessaloniki alerted for a more proactive response at the national level that resulted in a nationwide lockdown on 7th November. It seems that a similar pattern of epidemic could have evolved in Attika with a 10-day time difference but was interrupted by the 7th November lockdown. Indeed, the epidemic in Attika was decelerated, and the proportion of people aged >65 did not increase over the expected number of cases, resulting in lower morbidity and mortality.

Our study revealed the anatomy of the emergence of the SARS-CoV-2 second wave and explored the hypotheses of seasonal switching by showing the age and nighttime temperature pattern of emergence in two major metropolitan areas in Greece.

Our study has specific limitations that remain to be clarified to confirm our hypothesis. Most prominently an unbiased year-round study that would allow deeper understanding of the seasonal dynamics of SARS-CoV-2 was not possible until now. There are several reasons why such a unified year-round study could not be performed. Firstly, between March and May 2020 Greece was under a strict lockdown. Thus, data collected during this period was extremely limited and, crucially, cannot be compared with data collected later during the summer or autumn 2020. Between May 2020 and September 2020 incidence was very low as can be also seen in Figure 1: cases, patients in ICUs and daily deaths were extremely low, thus there is very limited information with respect to the virus circulation and the social mixing patterns. Since the low incidence continued within September and the first-third of October, we argue that the data presented here are sufficient to capture the switch that resulted into the sharp rise of SARS-CoV-2.

Most importantly indoor activities (bars, restaurants) were not allowed until at least end of July at which time point indoor entertainment was extremely limited due to high temperatures. A general lockdown (with curfew) was imposed at November 2021 as a result of the sharp rise of the cases and hospitalisations, thus the social mixing patterns were completely different. We have included 2 months after the lockdown to show how the mixing patterns changed. The vaccination program in Greece effectively started early in January 2021 and prioritized the vulnerable population (old, underlying chronic conditions) as well as the health professionals. This means that the study of transmission after January 2021 needs to consider the vaccination coverage that was largely different between age groups. Crucially, considering the effect of increasing vaccination in different age groups would require complex modeling and was beyond the scope of this hypothesis manuscript.

In December 2020 the first variants of concern were identified, namely Alpha, Beta and Gamma which significantly changed the epidemiology of the epidemic starting from January 2021. The Alpha variant dominated Greece from February until late Spring 2021. Thus, after January 2021 the epidemic was also affected by the gradual domination of the Alpha variant and thus cannot be compared to the previous epidemic profile. In March–April 2021 the Delta variant was identified in India. This resulted in increased transmissibility throughout summer and until December 2022 when the Omicron variant resulted in the largest increase of Covid-19 cases since the start of the epidemic.

**Figure 6:**
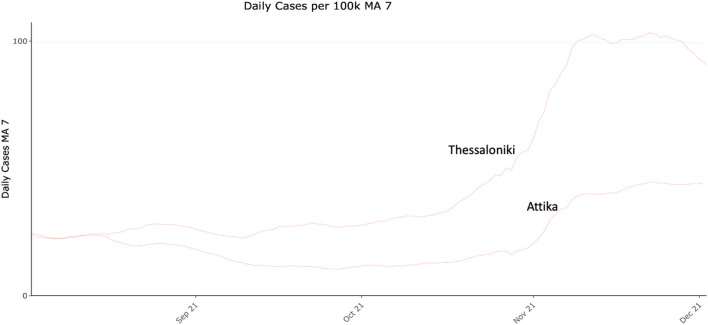
COVID-19 daily cases (7-day moving average) per 100.000 residents in Attika and Thessaloniki between August and December 2021.

Given the above-mentioned epidemiological dynamic changes, a year-round period of data could not provide unbiased data that would be informative for our hypothesis. In any case our presented comparison of the periods before the increased incidence as well as during the increased incidence is indicative of the transmission switch that drove increased transmissibility. Crucially, during October 2021 the increased transmissibility scenario was re-observed, firstly in Thessaloniki and then in Attika (Figure 6) similarly to 2020 in accordance with our hypothesis. However, comparability between these epidemic curves is confounded by differences in vaccination coverage between the two metropolitan areas at the time, thus not allowing a safe conclusion as with data collected in 2020. A continued monitoring of the situation over the following years will provide stronger evidence about the consistency of the pattern, and thus confirm the hypothesis.

Although it is likely that in different settings, the emergence of SARS-CoV-2 cold-season waves will be generated by other mechanisms, our hypothesis, if confirmed, has important implications for cold-season preparedness. First, the switch from outdoor to indoor entertainment activities over the cold seasons needs to be gradual and well-controlled. Second, a successful vaccination strategy should aim at increasing the vaccine coverage of the 18–39 age group. Even though they have lower morbidity and mortality (17, 18), their role in the social network is crucial, as they seem to be important drivers of the epidemic. In terms of immunity, this age group is likely to develop stronger and longer immune responses (19); thus, building an immune wall with certainty within this age group is likely feasible and durable. The desired coverage, however, needs to be at the highest possible level, probably higher than the average of the population, since their role in superspreading seems to be substantial. In light of our findings, vaccination of this age group might be important for decelerating cold-season dynamics of other respiratory diseases, such as influenza, a hypothesis that merits further exploration.

## Data Availability Statement

The original contributions presented in the study are included in the article/supplementary materials, further inquiries can be directed to the corresponding author/s.

## Author Contributions

SS and AB: acquisition of data. SS, AB, EJ, AK, PL, and GM: analyses and interpretation of data and finalizing the manuscript. GM: supervision and conceptualization. SS and GM: writing of first draft. All authors contributed to the article and approved the submitted version.

## Funding

GM's work was supported through a donation of SYN-ENOSIS, the Greek Shipowners' Social Welfare Company, to the Department of Hygiene, Epidemiology and Medical Statistics, National and Kapodistrian University of Athens.

## Conflict of Interest

The authors declare that the research was conducted in the absence of any commercial or financial relationships that could be construed as a potential conflict of interest.

## Publisher's Note

All claims expressed in this article are solely those of the authors and do not necessarily represent those of their affiliated organizations, or those of the publisher, the editors and the reviewers. Any product that may be evaluated in this article, or claim that may be made by its manufacturer, is not guaranteed or endorsed by the publisher.
